# Environmental determinants and risk behaviour in the case of indigenous malaria in Muara Enim Regency, Indonesia: A case-control design

**DOI:** 10.1371/journal.pone.0289354

**Published:** 2023-08-03

**Authors:** Hamzah Hasyim, Muhammad Aandi Ihram, Fakhriyatiningrum, Misnaniarti, Haerawati Idris, Iche Andriyani Liberty, Rostika Flora, Hilda Zulkifli, Zemenu Tadesse Tessema, Fadhilah Eka Maharani, Din Syafrudin, Patricia Dale

**Affiliations:** 1 Faculty of Public Health, Universitas Sriwijaya, Palembang, Indonesia; 2 Department of Public Health and Community Medicine, Faculty of Medicine, Universitas Sriwijaya, Palembang, Indonesia; 3 Department of Biology, Faculty of Mathematics and Natural Sciences, Universitas Sriwijaya, Palembang, Indonesia; 4 Department of Epidemiology and Preventive Medicine, School of Public Health and Preventive Medicine, Monash University, Melbourne, Australia; 5 Department of Epidemiology and Biostatistics, Institute of Public Health, College of Medicine and Health Sciences, University of Gondar, Gondar, Ethiopia; 6 Department Parasitology, Faculty of Medicine, Universitas Hasanuddin, Indonesia; 7 Centre for Planetary Health and Food Security (CPHFS) School of Environment, and Science, Griffith University, Nathan, Queensland, Australia; Menzies School of Health Research, AUSTRALIA

## Abstract

**Introduction:**

Malaria is a significant public health concern in Indonesia. Muara Enim Regency is one of the districts in South Sumatra with the most important number of indigenous malaria cases in the last three years (2018–2020). Therefore, this study aimed to identify determinants of indigenous malaria in the Muara Enim Regency.

**Methods:**

This study was designed as a case-control study. A stratified random sample in 2018, 2019, and 2020 was used at the Primary Health Centres (PHCs) areas of Tanjung Enim and Tanjung Agung. The sample included 49 cases and 49 controls. Indigenous malaria determinants were discovered using both bivariable and multivariable logistic regression models.

**Result:**

The multivariable logistic regression model results show that mosquito repellent reduces malaria risk by 71% (AOR = 0.29, 95% CI: 0.11–0.64). Besides, the presence of wire mesh on ventilation reduces the risk of malaria by 76% (AOR = 0.24, 95% CI: 0.10–0.57), and the distance from mosquito breeding sites near hundred meters and fewer increases the risk of malaria by 3.88 fold (AOR = 3.88; 95% CI: 1.67–8.97).

**Conclusions:**

Multivariable analysis revealed distance from mosquito breeding sites as a risk factor for malaria. Besides, the study shows that using insect repellent, wire netting in ventilation, eliminating mosquito breeding sites, mosquito repellent or protective clothing, and improving house conditions were protective factors for indigenous malaria. Therefore, preventive and promotional efforts are essential as the first step toward malaria elimination at the study site, including avoiding direct contact between residents and vectors near mosquito breeding sites.

## Introduction

Malaria is a public health concern and one of the world’s top causes of mortality; it is spread by the *Anopheles* mosquito, which carries the *Plasmodium* parasite. World Malaria Day 2023 reports 619,000 malaria fatalities and 247 million new cases in 2021 [1]. The third aim of the Sustainable Development Goals (SDGs) is to reduce malaria case incidence and mortality by 90% by 2030 [2].

The 2018 Riskesdas (Indonesia Basic Health Research) statistics for malaria incidence in Indonesia indicated that 10.7 million people continue to reside in medium and high malaria endemic areas. These territories include Papua, West Papua, and Nusa Tenggara Timur (NTT). In 2017, 266 districts/cities out of 514 in Indonesia were malaria-free, 172 districts/cities were low endemic, 37 districts/cities were medium endemic, and 39 districts/cities were severely endemic. Malaria prevalence in Indonesia is 0.37%. In South Sumatra, the malaria prevalence is 0.24% [3]. Malaria remains a public health issue in Indonesia, including in South Sumatra [4–8].

The Muara Enim Regency could be a breeding location for *Anopheles* mosquitoes. Certain areas are open mineral mining operations that may contain standing water and be a mosquito breeding risk. According to published studies, there is a variety of factors that can affect malaria risk. These include housing conditions, mosquito site locations; the presence of domestic animals; and community behaviour.

Based on Electronic System Information Surveillance Malaria (E-SISMAL), data on the Annual Parasite Incidence (API) in the study area over the previous five years. In 2018, the API decreased steadily at Primary Health Care (PHCs) of Tanjung Agung 0.7 and Tanjung Enim 0.83, followed by APIs of 0.49 and 0.31 in 2019 and 0.05 and 0.13 in 2020, respectively.

E-SISMAL reveals that the API in both PHCs dropped dramatically to zero in 2021. API in Tanjung Enim PHCs increased slightly to 0.03 in 2022. Muara Enim Regency had the most malaria cases in the past three years (2018–2020). PHCs of Tanjung Enim and Tanjung Agung had the most cases in their areas [9]. Most of these cases are local transmission. Five *Plasmodium* species cause malaria, which is spread by anopheline mosquitoes. Two of the five parasites that cause malaria in humans, *Plasmodium falciparum* and *Plasmodium vivax*, represent the most significant threat. In the study area, 22.45% of patients were infected with *Plasmodium falciparum*, 63.27% with Plasmodium vivax, and 14.28% with *Plasmodium mix*. The data can be accessed at http://sismal.malaria.id/. The data needs permission from the Ministry of Health of the Republic of Indonesia to access the dataset.

There are three leading indicators as absolute requirements to achieve malaria elimination, namely (1) API of less than 1 per 1000 population, (2) Slide Positive Rate of less than 5%, and (3) no indigenous cases for three consecutive years. Based on API, Muara Enim district has a low endemic status (with API <1). However, Muara Enim district is one of three regencies in South Sumatra with the highest malaria cases in the last three years. Indigenous issues are still found and have not yet achieved eliminated from 17 districts in South Sumatra Province.

World Health Organization (WHO) guidelines require no indigenous malaria cases before districts can be verified for malaria elimination. So, the research aimed to identify determinants of indigenous malaria in the Muara Enim Regency still found in the study site.

## Materials and methods

### Study area

This study was conducted in the service area in the PHCs of Tanjung Enim and Tanjung Agung. The PHcs were chosen based on the number of Indigenous malaria cases in Muara Enim Regency over three years (2018–2020). Malaria cases caused by something in the local area are called indigenous cases, contracted locally with no evidence of importation and no direct link to transmission from an imported case [10]. It is likely an indigenous case if the sufferer, during the incubation period (+/- 2 weeks), has no history of travelling to endemic areas.

**Fig 1:**
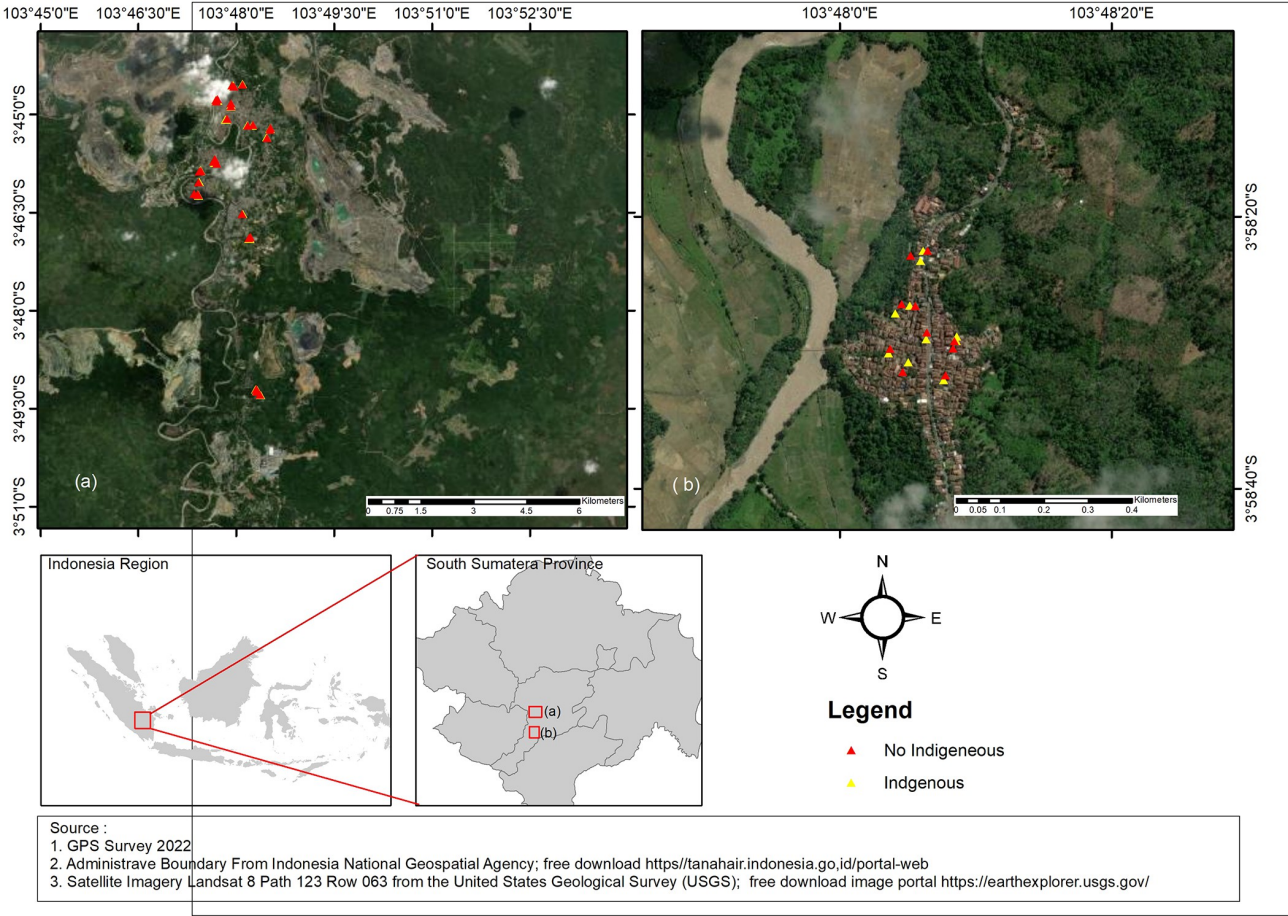
Study area.

The study area shows in Fig 1.

#### Research design

The case-control study design was carried out in May and June of 2022. This study used stratified random sampling to divide the population into groups using the year strata 2018, 2019, and 2020; simple random sampling was conducted from each stratum. The computation results yielded a total of 98 case and control samples. Respondents include indigenous malaria cases of all ages residing in two PHCs in Tanjung Enim and Tanjung Agung, recorded in E-SISMAL data in the malaria control program (2018–2020).

#### Estimated sample size

The sample size is calculated using the sample formula to test the two-proportion hypothesis [11].


$$n=\frac{{\left(Z_{1-\frac{a}{2}}\sqrt{2P(1-P)}+Z_{1-\beta }\sqrt{P_{1}(1-P_{1})+P_{2}(1-P_{2})}\right)}^{2}}{{\left(P_{1}-P_{2}\right)}^{2}}$$

It used P1 and P2 from previous studies for sample size and selected variables. This study used P1: the proportion of respondents with breeding place distance in the malaria group (the proportion of exposure in the case group) was 0.60, and P2: the proportion of respondents with breeding place distance in the non-malaria group (the proportion of exposure in the control group) was 0.31 [12]. The number of initial samples is calculated using the sample size program, with a significance level (95%), type I error (α) 5%, and test power (80%).

The stages of determining the minimum number of samples are carried out using the test two-proportion hypothesis. Then a stratified calculation was done using the year strata (2018–2020). We obtained a minimum sample size of (45). Then to avoid dropping out, 10% was added so that the minimum number of samples obtained was (49). Then, sample calculations were done using stratified random sampling in 2018–2020; This case-control design was carried out with a ratio of 1:1, so the total sample was (98) respondents. We used 49 cases and 49 controls. This calculation follows the method and research design used so that the minimum number of samples obtained represents the number of frames of the population in this area.

Then a sample search was conducted in the study area in nine selected villages in two sub-districts, namely Lawang Kidul and Tanjung Agung sub-districts, Muara Enim district, South Sumatra. After that, it was followed by tracing the control sample using individual matching "sex" on the three closest neighbours of the case. This technique uses a paper lottery with at least three of the nearest neighbours of the case and a random sampling system so that one number is selected as a control sample. Following the collection of case and control sample data, the investigation of malaria risk factors continues. All samples, both cases and controls, were conducted face-to-face interviews between the interviewer and the subject, using a valid and reliable structured questionnaire. The control subject was a healthy person who was the case’s nearest neighbour using sex-based matching.

Investigators used validity and reliability of the questionnaire were tested before data collection. The structured questionnaire has been tested for validity and reliability for 30 respondents whose characteristics are the same as the sample but carried out in neighbouring villages outside the study area. All participants, both cases and controls, were given the same valid and reliable questionnaire and equally administered. The questionnaire has been prepared to explore behavioural and environmental risk factors according to respondents’ experience, retrospectively, from 2018–2020, so the stages follow the case-control design flow.

It has been arranged to avoid information bias, and the questions asked to respondents are repeated so that the research subjects can understand and remember them.

#### Research variables

Indigenous malaria cases are the dependent variable. A case definition is an indigenous case who was positive for malaria based on data from the E-SISMAL in Muara Enim District (2018–2020). The dataset can be seen in the attachment.

Malaria sufferers are people infected with malaria parasites, as evidenced by the microscopic examination of malaria blood preparations. Health workers do microscopic exams to diagnose the diseases. Respondents who said they had never experienced malaria were asked about other illnesses-using questionnaires and E-SISMAL to validate interview disease data.

Independent variables included housing (the presence of wire netting, the condition of the floor of the house, and the condition of the house’s walls). Behavioural variables included mosquito nets, mosquito repellents, walking out at night, and wall-hanging garments.

Field observations revealed differences between respondents who lived in the urban village area and those who were in village areas; most respondents who were in the urban village area had the condition of floor of the house made of ceramic or cement. However, for respondents who are in village areas, there are still many stilt houses where the floors, walls and roofs are made of wood, and there are still holes or gaps for mosquitoes to enter the house, especially houses that are on the banks of rivers or areas near rice fields. Most respondents have a habit of hanging clothes on the house’s walls. These bad habits include hanging clothes on the bed, behind the bedroom door, in the window, on a chair, in the kitchen, and the bathroom.

Respondents were asked about using mosquito repellents by burning, rubbing and spraying mosquito coils and used at night before the respondent was diagnosed with indigenous malaria. It was also asked when to use it; if the respondent answered that he habitually used the repellent in a day, it was categorised as a protection variable. Researchers did not ask about the IRS (Indoor Residual Spraying) method.

All independent variables were recorded as one or two. Wire netting, breeding places, resting places, cow pens, and mosquito larvae are coded one if present and two if not; house floor and wall conditions are coded one if they do not meet standards and two if they do; breeding places and cattle pens are coded one if close and two if far. Regarding the breeding site in this study, we get the information through interviews and direct observation using GPS Essentials. Based on the literature review, the distance from mosquito breeding sites near hundred meters is categorised as close; otherwise, it is categorised far if there are no mosquito breeding places or within a radius of more than a hundred meters from the house [12]. Furthermore, cattle pens under ten meters define as close; otherwise categorised far if more than ten meters from the house [13]. In the current study using logistic regression, independent variables were normalised. They were all coded.

We selected backwards stepwise for multivariable regression models, which start with a saturated model and gradually reduce variables to find a reduced model that best explains the data.

### Ethics statement

On March 22, 2022, this research was approved by the health research ethics committee, Faculty of Public Health, Universitas Sriwijaya, number 312/UN9.FKM/TU.KKE/2022. There was no financial incentive. Before participation, the respondents provided written informed consent that all methods would follow relevant guidelines and regulations in the manuscript.

## Results

### Characteristics of the participants and demographics

Univariable analysis was used to examine each research variable, including behavioural factors, mosquito net use, mosquito repellent, being out of the house at night, and hanging clothes. Besides, Environmental factors follow wire mesh on ventilation, house floor condition, house wall condition, presence of mosquito breeding sites, presence of mosquito resting sites, mosquito breeding sites distance, the presence of the cattle cage, and the cattle cage distance. In addition, bivariable analysis was used to examine the associations between each behavioural and environmental factor with the occurrence of indigenous malaria. Univariable and bivariable analyses are exposed in Table 1.

**Table 1:** Univariable and bivariable analysis (n = 98).

Research variables	*Indigenous* Malaria		OR; 95% CI (lb-ub)	P-value
	Case (%)	Control (%)		
**Using Mosquito Net**				
No	64.7	35.3	0.28 (0.12–0.64)	0.005
Yes	34.0	66.0		
**Using Mosquito Repellent**				
No	32.6	67.4	0.27 (0.11–0.64)	0.004
Yes	63.6	36.4		
**Out of the House at Night**				
Yes	56.8	43.2	1.54 (0.68–3.52)	0.405
No	45.9	54.1		
**Hanging Clothes**				
Yes	54.4	39.5	2.58 (0.89–7.49)	0.125
No	31.6	68.4		
**Wire Mesh on Ventilation**				
No	30.0	70.0	0.24 (0.10–0.57)	0.002
Yes	63.8	36.2		
**House Floor Condition**				
Not eligible	57.1	42.9	1.44 (0.54–3.81)	0.622
Qualify	48.1	51.9		
**House Wall Condition**				
Not eligible	60.0	40.0	1.72 (0.68–4.33)	0.354
Qualify	46.6	53.4		
**Mosquito Breeding Sites**				
There is	61.8	38.2	3.02 (1.31–6.93)	0.015
There is not	34.9	65.1		
**Mosquito Resting Sites**				
There is	61.2	38.8	2.49 (1.10–5.61)	0.043
There is not	38.8	61.2		
**Mosquito Breeding Distance**				
Near 100m	66.7	33.3	3.88 (1.67–8.97)	0.002
Far >100 m	34.0	66.0		
The Presence of the Cattle Cage				
There is not	43.9	56.1	0.55 (0.24–1.24)	0.219
There is	58.5	41.5		
**Cattle Cage Distance**				
Near 10m	55.6	44.4	1.42 (0.62–3.24)	0.53
Far >10 m	46.8	53.2		
**Mosquito Larvae**				
There is	44.1	55.9	0.69 (0.30–1.60)	0.524
There is not	53.1	46.9		

*lb* Lower 95% confidence boundary of cell per cent age, *ub* Upper 95% confidence boundary of cell per cent age

Table 1 shows that six variables significantly differed between case and control subjects. The association indicated some relevance to malaria risk. The connections are noted next for these six variables. They were: net use (64.7% of cases did not use nets; 66% of the controls did); Use of mosquito repellents (63.6 of cases did have; 67.4 of the controls did not; having wire mesh on ventilation (63.8% of cases did have; 70% of controls did not); the existence of mosquito breeding sites (61.8% of cases did have; 65.1% of controls did not);: Distance to mosquito breeding sites nearby (100m) (66.7% of cases were near breeding places, 66% of controls were not near, were > 100 m away). Bivariable analysis revealed a correlation between the use of mosquito nets (p-value 0.005), the use of mosquito repellent (p-value 0.004), the presence of wire netting in ventilation (p-value 0.002), the presence of breeding places around the house (p-value 0.015), the presence of resting places around the house (p-value 0.043), and the distance between the mosquito breeding sites and the house (p-value 0.002) for cases of indigenous malaria in the study area.

Furthermore, a multivariable analysis was performed to identify the predominant risk factors for instances of Indigenous malaria, shown in Table 2.

**Table 2:** Multivariable analysis.

Research variables	Simple logistic regression analysis		Multiple logistic regression analysis	
	OR (95% CI)^a^	P-value	AOR (95% CI)^b^	P-value
Using Mosquito Repellent				
No	1		1	
Yes	0.28 (0.08–0.90)	0.035	0.29 (0.09–0.91)	0.035
***wire mesh*** screen on ventilation				
Yes	0.17 (0.05–0.62)	0.007	0.18 (0.05–0.62)	0.007
No	1		1	
Breeding sites				
Near 100m	3.54 (1.12–11.1)	0.031	3.45 (1.11–10.7)	0.032
Far >100 m	1		1	

Ref.: The reference category is represented in the contrast matrix as a row of one

^a^Crude odds ratio (OR)

^b^Adjusted odss ratio (AoR)

The multivariable logistic regression model results show that mosquito repellent reduces malaria risk by 71% (OR = 0.29, 95% CI: 0.09–0.91). Besides, the presence of wire mesh on ventilation reduces the risk of malaria by 76% (OR = 0.18, 95% CI 0.05–0.62), and the distance from mosquito breeding sites near hundred meters and fewer increases the risk of malaria by 3.45 fold (OR = 3.45; 95% CI 1.11–10.7).

## Discussion

It is interesting to study indigenous malaria in low-endemic areas. This study revealed that the multivariable logistic regression model results show mosquito repellent reduces malaria risk. Besides, wire mesh in ventilation reduces the risk of malaria. Another hand, the distance from mosquito breeding sites is nearly a hundred meters, or less increases the risk of malaria.

This research discovered that mosquito repellent reduces malaria risk. In line with previous research, they have demonstrated that using an insect repellent will protect humans from malaria-spreading mosquitoes. A study in Ethiopia looked at insect/mosquito repellent plants used by 97.2% of respondents. Hamlets have traditionally used insect/mosquito-repellent plants [14]. Another study found a need to improve *Plasmodium* infection prevention and control strategies, specifically by increasing personal mosquito repellent [15]. In another study, mosquito coils did not diminish malaria transmission. Repellent distribution and exchange had higher operational expenses than long-lasting insecticidal nets (LLIN) distribution [16]. Such effort could be acceptable in the context of malaria elimination, where these interventions are temporary [17].

This study revealed the importance of the presence of mosquito breeding sites. Mosquito breeding sites near homes are common in sub-Saharan Africa and cause malaria infection [18]. In Kenya, malaria prevalence was strongly correlated with the density of mosquito breeding sites [19]. Therefore, it was recommended that mosquito breeding places be eliminated [20]. Understanding the feeding and resting habits and the transmission potential of adult vectors in the area is crucial for proper planning and implementing enhanced vector control strategies [21]. Natural or man-made water bodies, large or small, in rushing or stagnant water, shady or sunny, permanent or transitory. Variations in *Anopheles* densities affect malaria risk spatially and temporally [22].

This study demonstrated that the presence of wire mesh in ventilation reduces the risk of malaria. Similarly, wire mesh screens are a practical way to reduce exposure to malaria-transmitting insects. Screening protects indoor sleepers from mosquito-borne infections [23]. House improvements included covering eaves, other wall gaps, and doorways with locally available materials and screening ventilation holes/spaces, including windows, with wire mesh [24].

Similarly, housing improvements offer a viable strategy for vector control in low-transmission environments that may impart a similar protective benefit to current vector control strategies [25]. Good housing construction limits mosquito vector access, reducing malaria risk. Ugandan housing design affects mosquito ingress and malaria risk [26].

## Limitations of research

E-SISMAL data determined malaria disease status retrospectively, not researchers’ diagnoses. Thus, only respondents with professional malaria diagnoses could determine malaria prevalence. E-SISMAL did not record all factors affecting malaria transmission in the study area. Future research may address these. E-SISMAL, a WHO-recommended malaria surveillance method, and a structured questionnaire benefit this study. The findings suggest indigenous case risk variables could be used to design malaria control programs in low-endemic areas.

## Conclusions

Mosquito repellent, mosquito breeding sites distance, and wire mesh on ventilation are determinants of indigenous malaria in Muara Enim Regency. Eliminating malaria in the study site requires boosting promotional efforts in mosquito prevention measures. For suggestions for malaria protection, the community is urged to carry out malaria vector control: Environmental management in the form of Stockpiling or drying water drainage and cleaning aquatic plants. Spreading fish that eat mosquito larvae/larvae Using mosquito nets, Reducing activities outside he house at night to avoid Anopheles mosquito bites, and Sprinkle larvicide powder, Install wire gauze/wire mesh.

## Supporting information

S1 File (ZIP)

## Abbreviations

API: Annual Parasite Incidence
AOR: Adjusted odds ratio
CI: confident interval
E-SISMAL: Electronic System Information Surveillance
LLIN: Long-Lasting Insecticidal Nets; Malaria
Kemenkes: Ministry of Health
Nusa Tenggara Timur (NTT): East Nusa Tenggara
PHCs: Primary Health Cares
RISKESDAS: Indonesia Basic Health Research
SDGs: Sustainable Development Goals
WHO: World Health Organization
